# Assessing the Impact of I_h_ Conductance on Cross-Frequency Coupling in Model Pyramidal Neurons

**DOI:** 10.3389/fncom.2020.00081

**Published:** 2020-09-10

**Authors:** Melvin A. Felton, Alfred B. Yu, David L. Boothe, Kelvin S. Oie, Piotr J. Franaszczuk

**Affiliations:** ^1^Combat Capabilities Development Command (CCDC)—Army Research Laboratory, Adelphi, MD, United States; ^2^Combat Capabilities Development Command (CCDC)—Army Research Laboratory, Aberdeen Proving Ground, MD, United States; ^3^Department of Neurology, Johns Hopkins University School of Medicine, Baltimore, MD, United States

**Keywords:** cross-frequency coupling, phase-amplitude coupling, theta-gamma coupling, pyramidal neuron, I_*h*_ conductance

## Abstract

Large cortical and hippocampal pyramidal neurons are elements of neuronal circuitry that have been implicated in cross-frequency coupling (CFC) during cognitive tasks. We investigate potential mechanisms for CFC within these neurons by examining the role that the hyperpolarization-activated mixed cation current (I_h_) plays in modulating CFC characteristics in multicompartment neuronal models. We quantify CFC along the soma-apical dendrite axis and tuft of three models configured to have different spatial distributions of I_h_ conductance density: (1) exponential gradient along the soma-apical dendrite axis, (2) uniform distribution, and (3) no I_h_ conductance. We simulated two current injection scenarios: distal apical 4 Hz modulation and perisomatic 4 Hz modulation, each with perisomatic, mid-apical, and distal apical 40 Hz injections. We used two metrics to quantify CFC strength—modulation index and height ratio—and we analyzed CFC phase properties. For all models, CFC was strongest in distal apical regions when the 40 Hz injection occurred near the soma and the 4 Hz modulation occurred in distal apical dendrite. The strongest CFC values were observed in the model with uniformly distributed I_h_ conductance density, but when the exponential gradient in I_h_ conductance density was added, CFC strength decreased by almost 50%. When I_h_ was in the model, regions with much larger membrane potential fluctuations at 4 Hz than at 40 Hz had stronger CFC. Excluding the I_h_ conductance from the model resulted in CFC either reduced or comparable in strength relative to the model with the exponential gradient in I_h_ conductance. The I_h_ conductance also imposed order on the phase characteristics of CFC such that minimum (maximum) amplitude 40 Hz membrane potential oscillations occurred during I_h_ conductance deactivation (activation). On the other hand, when there was no I_h_ conductance, phase relationships between minimum and maximum 40 Hz oscillation often inverted and occurred much closer together. This analysis can help experimentalists discriminate between CFC that originates from different underlying physiological mechanisms and can help illuminate the reasons why there are differences between CFC strength observed in different regions of the brain and between different populations of neurons based on the configuration of the I_h_ conductance.

## Introduction

Cross-frequency coupling (CFC) has been associated with mental processes like perceptual and memory-related tasks, and is often observed via electroencephalogram (EEG) and local field potential (LFP) measurements (Jensen and Colgin, 2007; Tort et al., 2009; Canolty and Knight, 2010; Lisman and Jenson, 2013). Different types of network properties can yield distinct CFC signatures [see Hyafil et al. (2015) for a review], and there are a variety of physiological mechanisms believed to contribute to CFC in pyramidal neurons, such as the timing of upstream inputs (Fernández-Ruiz et al., 2017), or fast synaptic inhibition (Wuff et al., 2009), and NMDA-mediated excitation of related interneuronal populations (Korotkova et al., 2010). However, the detailed nature of the role that intracellular mechanisms play in CFC of pyramidal neurons is still uncertain.

The way individual pyramidal neurons, and therefore the neuronal networks they are a part of, respond to synaptic input in particular frequency ranges is largely governed by ion channels (Lai and Jan, 2006; Nusser, 2012). In particular, the hyperpolarization-activated mixed cation current (I_h_) plays a multitude of roles in the regulation of neuronal and network excitability impacting both membrane resting potential and rhythmic activity, as well as the magnitude of excitatory post-synaptic potentials (EPSPs) (Nusser, 2009; Brennan et al., 2016). The different effects of the I_h_ conductance suggest that it may play an important role in the occurrence of CFC within individual pyramidal neurons, however this remains uncertain. Vaidya and Johnston (2013) report gamma-theta correlation of synaptic currents observed in single CA1 pyramidal neurons that is distinct from the phenomenon of cross-frequency phase coupling observed at the network level. In addition, using a model of hippocampal CA3, Neymotin et al. (2013) showed that the density of pyramidal neuron I_h_ conductance modulated the amplitude of CFC observed in the simulated LFP (network-level CFC). Because there are differences in the subcellular distribution of I_h_ in distinct classes of pyramidal neurons (Bullis et al., 2006; Nusser, 2009), I_h_ may have a variable impact on CFC depending upon factors like regional specialization or underlying pathological conditions.

As an extension of our previous work examining coupling between perisomatic and distal apical functional zones in cortical layer five pyramidal neurons (Felton et al., 2018), we examined CFC along the soma-apical dendrite axis and tuft of realistic compartmental models of large pyramidal neurons. In particular, our baseline model neuron possessed an exponentially-increasing gradient of I_h_ conductance density along the apical dendrite like cortical layer five and hippocampal CA1 pyramidal neurons are known to possess (Hu et al., 2009; Nusser, 2009; Hay et al., 2011). To assess the role of the I_h_ conductance in the occurrence of CFC within neocortical and limbic pyramidal neurons, we configured a total of three models, each with the same morphology, but with different spatial distributions of I_h_ conductance density. We also examine the effect on CFC of fundamentally different input configurations to large pyramidal neurons. We simulated two modulation scenarios, one based on distal 4 Hz modulation and the other on perisomatic 4 Hz modulation. For each of these modulation types, 40 Hz current injections were simulated in key locations throughout the apical dendrite, namely in perisomatic, middle apical, and distal apical compartments. We used two metrics to quantify the strength of CFC—modulation index and height ratio (Tort et al., 2010)—and we analyzed the phase properties of CFC.

## Methods

### Models

For this study, we adapted the model of a large cortical pyramidal neuron used in Felton et al. (2018). This model was obtained by modifying the regular-spiking, layer five pyramidal neuron model used by Traub et al. (2005), so that it possessed characteristics now known to be common among this class of neuron. The characteristics of this model included an exponentially increasing gradient of the I_h_ conductance density ascending along the soma-apical dendrite axis, and a distal apical Ca^2+^ hot zone where the conductance densities for the high-threshold and low-threshold Ca^2+^ channels are 10 and 100 times higher, respectively, than anywhere else in the apical dendrite and tuft (Hay et al., 2011). However, our focus in the current study is primarily on the impact of I_h_ on CFC. Therefore, the conductance densities for the two Ca^2+^ conductances were reverted back to Traub et al. (2003, 2005).

To evaluate the impact of the I_h_ conductance on CFC, we evaluated three models distinguished by the configuration of the I_h_ conductance throughout the neuronal membrane. The first model had a somato-apical dendritic exponential gradient in the I_h_ conductance common to many pyramidal neurons in layer five of the cortex or in the hippocampus (Hu et al., 2009; Nusser, 2009; Hay et al., 2011). The second model had almost an entirely uniform I_h_ conductance density distribution which can be found in various neuron types throughout the cortex, and in some cases, a uniformly-distributed I_h_ conductance can underlie pathological conditions like epilepsy (Nusser, 2009; Brennan et al., 2016). The third model did not have an I_h_ conductance, which may not be common at all in the cortex or hippocampus for large pyramidal neurons, but can be induced with the use of an I_h_ channel blocker, such as 4-(N-ethyl-N-phenylamino)-1, 2-dimethyl-6-(methylamino) pyrimidinium chloride (ZD7288) (Zhang et al., 2016). See Table 1 for detailed ionic conductance density configuration for each of these three models.

**Table 1:** Membrane conductance densities (mS/m^2^) by level in the model structure for the three model pyramidal neurons.

**Level**	**g_**Na(F)**_**	**g_**Na(P)**_**	**g_**K(DR)**_**	**g_**K(C)**_**	**g_**K(A)**_**	**g_**K(M)**_**	**g_**K2**_**	**g_**Ca(H)**_**	**g_**Ca(L)**_**	**g_h_exp_**	**g_h_unif_**	**g_h_0_**
0	0	5	450	0	6	600	15	0	0	0	0	0
1	0	5	450	0	6	300	15	0	0	0	0	0
2	0	5	750	288	200	250	15	16	1	1	1	0
3	0	0.6	250	288	40	136	0	16	1	1.7635	1	0
4	0	0.12	0	9	40	136	0	16	1	2.4515	1	0
5	0	0.12	0	9	40	136	0	16	1	3.3194	1	0
6	0	1.2	500	288	40	136	15	16	1	1.7635	1	0
7	0	0.6	150	288	40	136	15	16	1	2.4515	1	0
8	0	0.12	2	9	40	136	15	16	1	3.3194	1	0
9	0	0.12	2	9	40	40	0	4	1	4.4141	1	0
10	0	0.12	2	9	40	40	0	4	1	5.7950	1	0
11	0	0.12	2	9	40	40	0	4	1	7.5368	1	0
12	0	0.12	2	9	40	40	0	4	1	9.7338	1	0
13	0	0.12	2	9	40	40	0	4	1	12.5050	1	0
14	0	0.12	2	9	40	40	0	4	1	16.0006	1	0
15	0	0.024	2	9	40	40	0	4	1	20.4098	1	0
16	0	0.024	2	9	40	10	10	4	1	25.9714	1	0
17	0	0.024	2	9	40	10	10	4	1	32.9866	1	0
18	0	0.024	2	9	40	10	10	4	1	41.8355	1	0
19	0	0.024	2	9	40	10	10	10.8	1	52.9971	2	0
20	0	0.024	2	9	40	10	10	2.4	1	52.9971	2	0

*I_h_ columns are highlighted in maroon because their conductance density values were varied to obtain the three scenarios considered in this study: g_h_exp_- exponential gradient (Hay et al., 2011), g_h_unif_- uniform (Traub et al., 2005), g_h_0_- no I_h_. Explanation of levels: level 0 = initial axon; level 1 = rest of axon; level 2 = soma; level 3 = proximal basal and oblique dendrites; level 4 = middle basal and oblique dendrites; level 5 = distal basal and oblique dendrites; levels 6–18 = progressively more distal apical dendrite shaft (apdend1 to apdend13); level 19 = proximal apical tuft; level 20 = distal apical tuft*.

### CFC Analysis

**Figure 1:**
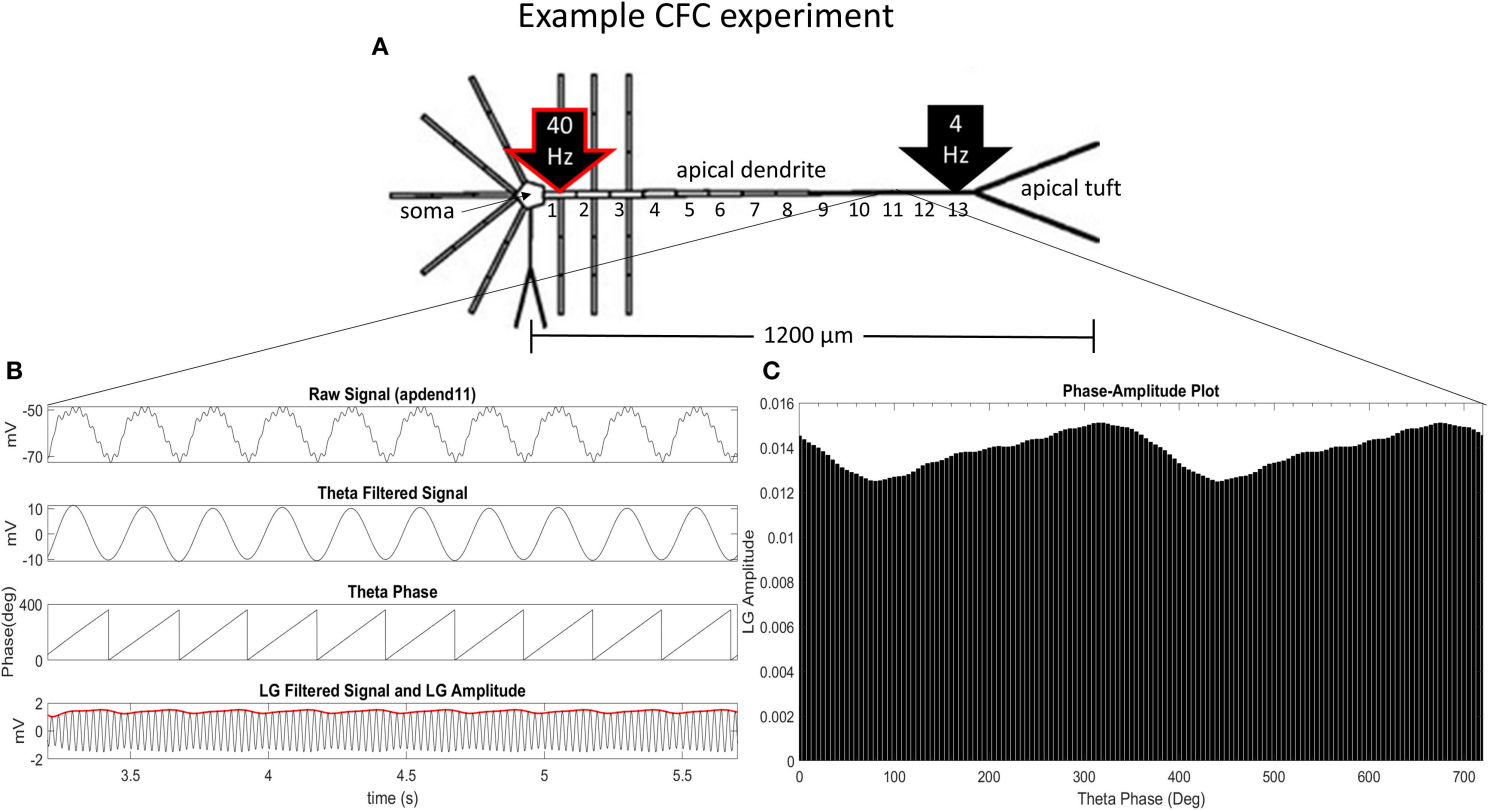
CFC analysis performed on model pyramidal neuron. **(A)** A 1.5 nA 40 Hz sinusoidal current was injected into the base of the apical dendrite while a 1.5 nA 4 Hz sinusoidal current was injected into the distal apical dendrite. Additonal labels include: soma, apical dendrite, and apical tuft; apical dendrite compartments 1–13 (apdend1, apdend2, …, apdend13); and the distance from soma to end of apical tuft−1,200 μm. **(B)** From top to bottom: membrane potential oscillations in the 11th distal apical dendrite compartment (apdend11) resulting from the 40 Hz and 4 Hz current injections, filtered 4 Hz component of the membrane potential oscillation, time series of 4 Hz phase, and filtered 40 Hz component of membrane potential oscillation (black) and amplitude envelope of 40 Hz oscillation (red). **(C)** Phase-amplitude plot for two complete 4 Hz cycles with a phase bin size of 5° (LG–low gamma, ~40 Hz).

Our CFC quantification analysis was based on the work of Tort et al. (2010). In our study, we focus on theta-gamma phase-amplitude coupling within large pyramidal neurons, which is commonly observed in cortex and hippocampus (Tort et al., 2010). Figure 1A illustrates an example simulation scenario where CFC was induced by injecting a 40 Hz sinusoidal current at the base of the apical dendrite while a 4 Hz sinusoidal current was injected in the distal apical dendrite. Figures 1B,C show several diagnostic plots that result from the CFC quantification analysis, in this case, for the 11th apical dendrite compartment from the soma (distal).

Following Tort et al. (2010), we obtain the diagnostic plots in Figure 1B that, from top to bottom, show the unfiltered membrane potential oscillations for the compartment that result from the two current injections, the filtered 4 Hz membrane potential oscillation for the compartment, the time series of the 4 Hz oscillation phase for the compartment, and the amplitude envelope (red) and filtered 40 Hz oscillation for the compartment (black). In this example, the simulation was run on the model with an exponential gradient in I_h_ conductance density.

To obtain the phase-amplitude plot from which the two CFC metrics used in this analysis are calculated, we first bin the phases of 4 Hz oscillations over the time period of analysis, and then calculate the mean of the 40 Hz oscillation amplitude envelope within each phase bin, *j*, denoted by 〈*A*_40_〉(*j*). After normalizing by the sum over all phase bins (*N*), we obtain the following expression for the normalized amplitude (*P*) distribution:

(1) $$\begin{array}{r} P\left(j\right)=\;\frac{\langle A_{40}\rangle \left(j\right)}{\sum_{k=1}^{N}\langle A_{40}\rangle \left(k\right)} \end{array}$$

Figure 1C is a phase-amplitude plot that is obtained by plotting *P* as a function of phase bin. For our analysis, we chose *N* = 72 to obtain high-resolution CFC phase information (5° bins of the 4 Hz signal phase).

We use two metrics to quantify CFC. We calculate a modulation index, *MI*, introduced by Tort et al. (2010). With values between 0 and 1, this metric quantifies the distance between the normalized amplitude distribution, *P*, and the uniform distribution, or, the case when there is no CFC.

(2) $$\begin{array}{r} MI=\;\frac{\log N+\;\sum_{j=1}^{N}P\left(j\right)\log P\left(j\right)}{\log N} \end{array}$$

The second metric we use to quantify CFC is height ratio, which is defined as:

(3) $$\begin{array}{r} height\;ratio=\;\frac{h_{max}-h_{min}}{h_{max}} \end{array}$$

where *h*_*max*_ and *h*_*min*_ are the maximal and minimal normalized amplitudes, respectively, in the phase-amplitude plot (Figure 1C). The *MI* and height ratio values for the phase-amplitude plot in Figure 1C are 3.97 × 10^−4^ and 0.1735, respectively.

### Sensitivity Analysis: Number of Bins and Injection Current Amplitude

**Figure 2:**
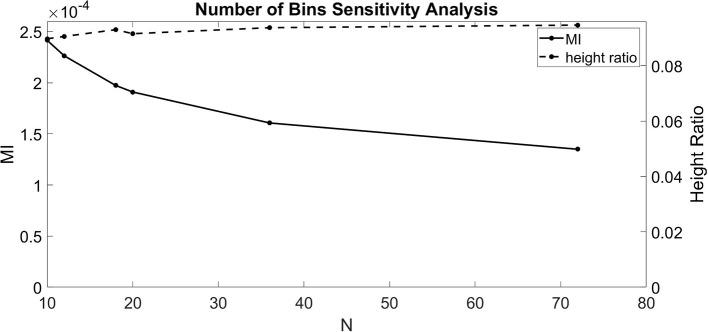
Sensitivity analysis for the number of bins (*N*). MI index (solid, left ordinate) and height ratio (dashed, right ordinate) as a function of *N*. Values for *N* evaluated (10, 12, 18, 20, 36, and 72) indicated by dots.

The dependence of Equations (1–3) on *N* indicates that the number of bins chosen affects the calculated value for MI and height ratio. To better understand these dependencies, we ran the CFC analysis for several values of *N* (10, 12, 18, 20, 36, and 72). For this test, we injected a 1.5 nA 4 Hz sinusoidal current into the soma and a 1.5 nA 40 Hz sinusoidal current into the base of the apical dendrite in the model with an exponential gradient in I_h_ conductance density along the soma-apical dendrite axis. Over the range of values for *N* considered, MI decreased monotonically with some flattening as *N* increased (Figure 2). By contrast, height ratio remained mostly constant, particularly as the value of *N* increased (Figure 2). This result suggests that height ratio is the CFC metric that can more readily be compared across experiments that use different bin sizes. For this reason, we use height ratio as the CFC metric to present the results of this study. The corresponding MI values are presented in Figures S1, S2. For reference, Tort et al. (2010) determined that the significance threshold for concluding if CFC is present is MI = 4.30 × 10^−5^ (number of bins *N* = 18).

**Figure 3:**
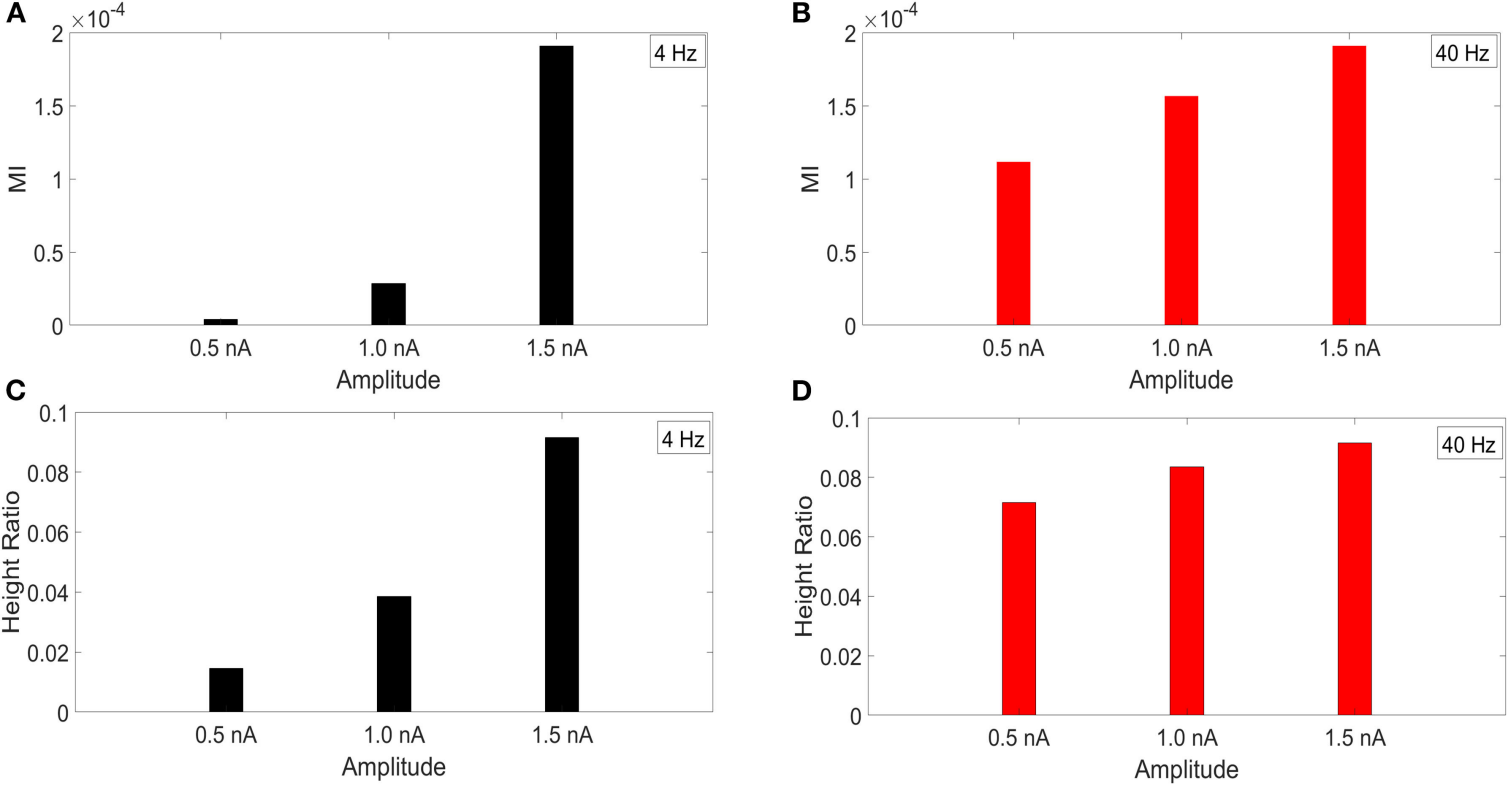
Current injection amplitude sensitivity analysis. **(A,C)** MI and height ratio, respectively, for amplitudes of the 4 Hz current injection of 0.5, 1.0, and 1.5 nA while the amplitude of the 40 Hz injection was held constant at 1.5 nA. **(B,D)** MI and height ratio, respectively, for amplitudes of the 40 Hz current injection of 0.5, 1.0, and 1.5 nA while the amplitude of the 4 Hz injection was held constant at 1.5 nA.

It has been reported that the amplitude of the membrane potential oscillations involved in CFC can affect the strength of CFC. In particular, this has been observed for slow frequencies, such as delta (0.5–4 Hz), theta (4–8 Hz), and alpha (8–12 Hz) (Hyafil et al., 2015). To examine this effect, we performed an amplitude sensitivity test for both the 4 and 40 Hz current injections (Figure 3) in the model with a soma-apical dendrite exponential gradient in I_h_ conductance density. In one scenario, we kept the amplitude of the 40 Hz current injection into the base of the apical dendrite constant at 1.5 nA while we performed the CFC analysis three times for 4 Hz somatic current injections with amplitudes of 1.5, 1.0, and 0.5 nA. Similarly, we ran the CFC analysis with constant 4 Hz amplitude of 1.5 and 40 Hz amplitudes of 1.5, 1.0, and 0.5 nA.

For both MI and height ratio, differences in amplitude of the 4 Hz current injection had a much bigger impact on CFC strength. Because we employ a variety of injection scenarios and there is a differential degree of filtering for the 4 and 40 Hz oscillations as they spread from their respective injection sites, the results of this amplitude sensitivity test will be useful for interpreting the CFC observed in our simulations.

## Results

### Two Injection Scenarios

We used two injection scenarios to test for a range of possible input configurations of large cortical and limbic pyramidal neurons. In the first injection scenario, we simulated a 1.5 nA 4 Hz sinusoidal current injection into the most distal apical dendrite compartment while a 1.5 nA 40 Hz sinusoidal current was injected into a perisomatic (base of apical dendrite), middle apical, and distal apical dendrite compartment on successive runs (not simultaneous). This injection scenario is consistent with slow distal modulation of cortical pyramidal neurons via matrix thalamocortical or higher-order feedback (VanRullen and Koch, 2003; Spruston, 2008; Larkum, 2013).

A second injection scenario simulates perisomatic 4 Hz modulation. A 1.5 nA 4 Hz sinusoidal current was injected into the soma while a 1.5 nA 40 Hz sinusoidal current was injected into a perisomatic, middle apical, and distal apical dendrite compartment on successive runs. This type of slow modulation of the soma can be mediated by parvalbumin immunoreactive interneurons (PV) in the hippocampus and cortex (Stark et al., 2013).

### CFC Strength: Distal 4 Hz Modulation

**Figure 4:**
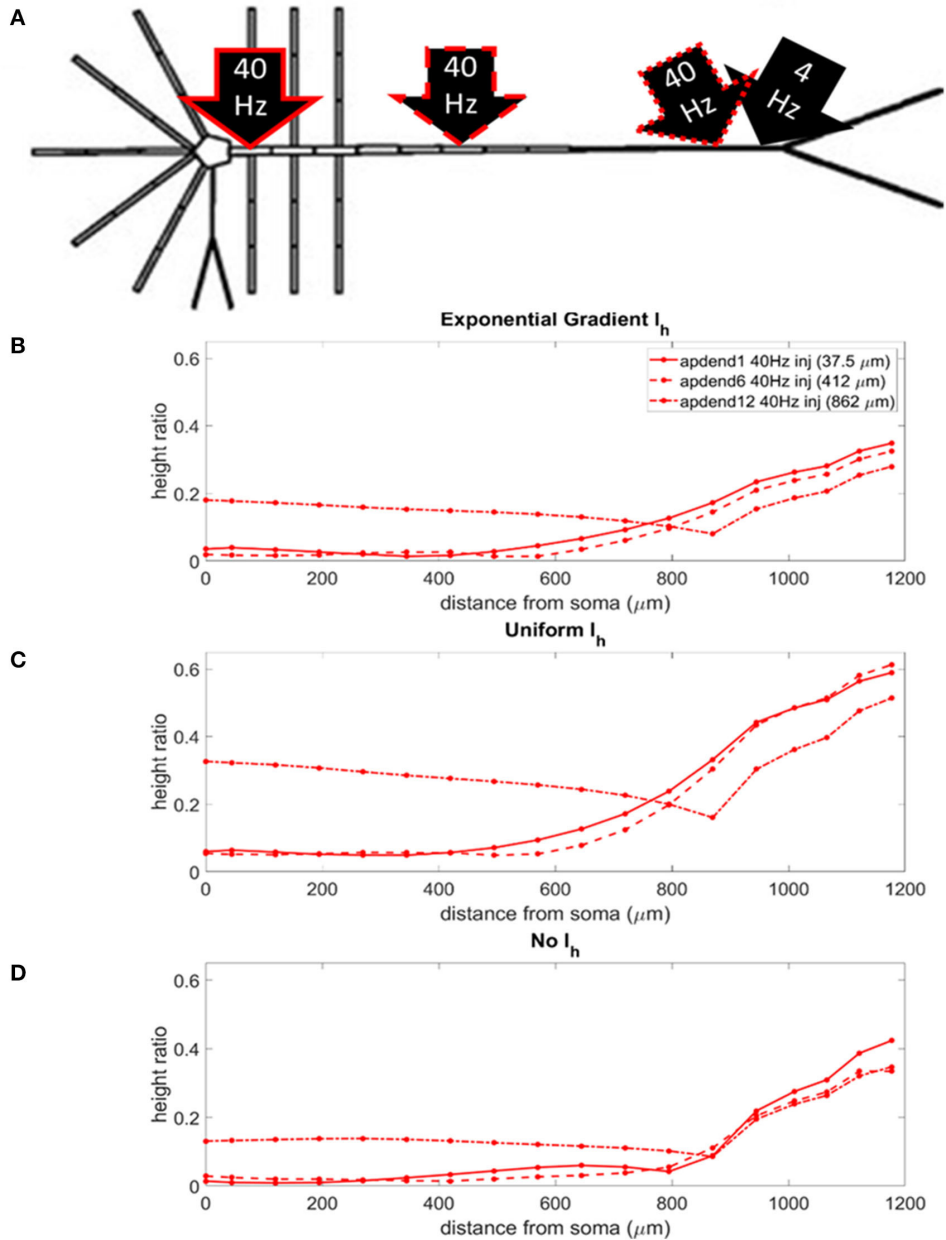
**(A)** Distal apical dendrite 1.5 nA, 4 Hz modulation (black arrow) with 1.5 nA 40 Hz current injections in base of apical dendrite (black arrow, solid red outline), middle apical dendrite (black arrow, dashed red outline), and distal apical dendrite (black arrow, dot-dashed red outline). **(B)** Height ratio calculated for the soma, apical dendrite, and apical tuft in the model with exponential gradient in I_h_ conductance density along apical dendrite. **(C)** Height ratio calculated for the soma, apical dendrite, and apical tuft in model with uniform I_h_ conductance density. **(D)** Height ratio calculated for the soma, apical dendrite, and apical tuft in model with no I_h_ conductance. Dot symbols along the profiles in **(B–D)** indicate the distance from the soma of compartments along the soma-apical dendrite axis and apical tuft (measured from the beginning of each compartment).

**Figure 5:**
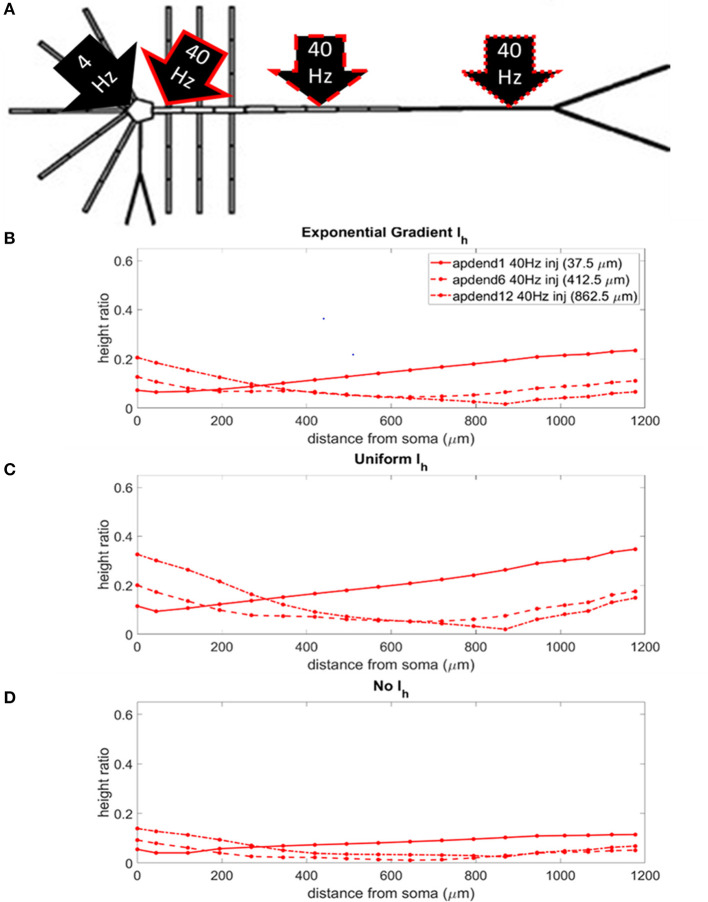
**(A)** Perisomatic 1.5 nA, 4 Hz modulation (black arrow) with 1.5 nA 40 Hz current injections in base of apical dendrite (black arrow, solid red outline), middle apical dendrite (black arrow, dashed red outline), and distal apical dendrite (black arrow, dot-dashed red outline). **(B)** Height ratio calculated for the soma, apical dendrite, and apical tuft in model with exponential gradient in I_h_ conductance density along apical dendrite. **(C)** Height ratio calculated for the soma, apical dendrite, and apical tuft in model with uniform I_h_ conductance density. **(D)** Height ratio calculated for the soma, apical dendrite, and apical tuft in model with no I_h_ conductance. Dot symbols along the profiles in **(B–D)** indicate the distance from the soma of compartments along the soma-apical dendrite axis and apical tuft (measured from the beginning of each compartment).

**Figure 6:**
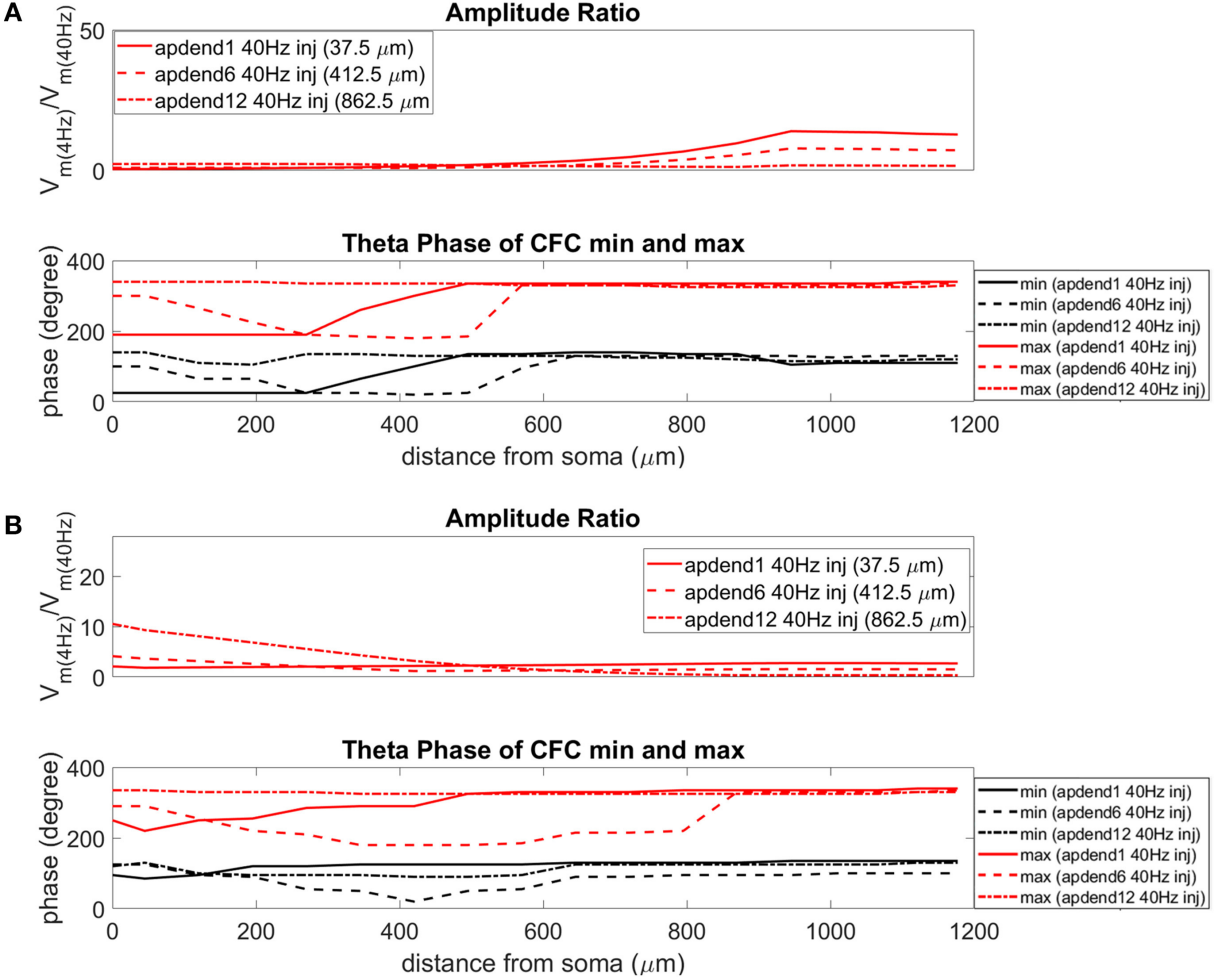
Amplitude ratio and theta phase of CFC for model with exponential gradient in I_h_ conductance. **(A)** Distal 4 Hz modulation, amplitude ratio (top), and theta phase (bottom) of minimum (black) and maximum (red) amplitude of 40 Hz membrane potential oscillation. Forty Hertz current injected into base of apical dendrite (solid lines), middle of apical dendrite (dashed lines), and distal apical dendrite (dot-dashed lines). **(B)** Perisomatic 4 Hz modulation, amplitude ratio (top) and theta phase (bottom) of minimum (black) and maximum (red) amplitude of 40 Hz membrane potential oscillation. Forty Hertz current injected into base of apical dendrite (solid lines), middle of apical dendrite (dashed lines), and distal apical dendrite (dot-dashed lines).

**Figure 7:**
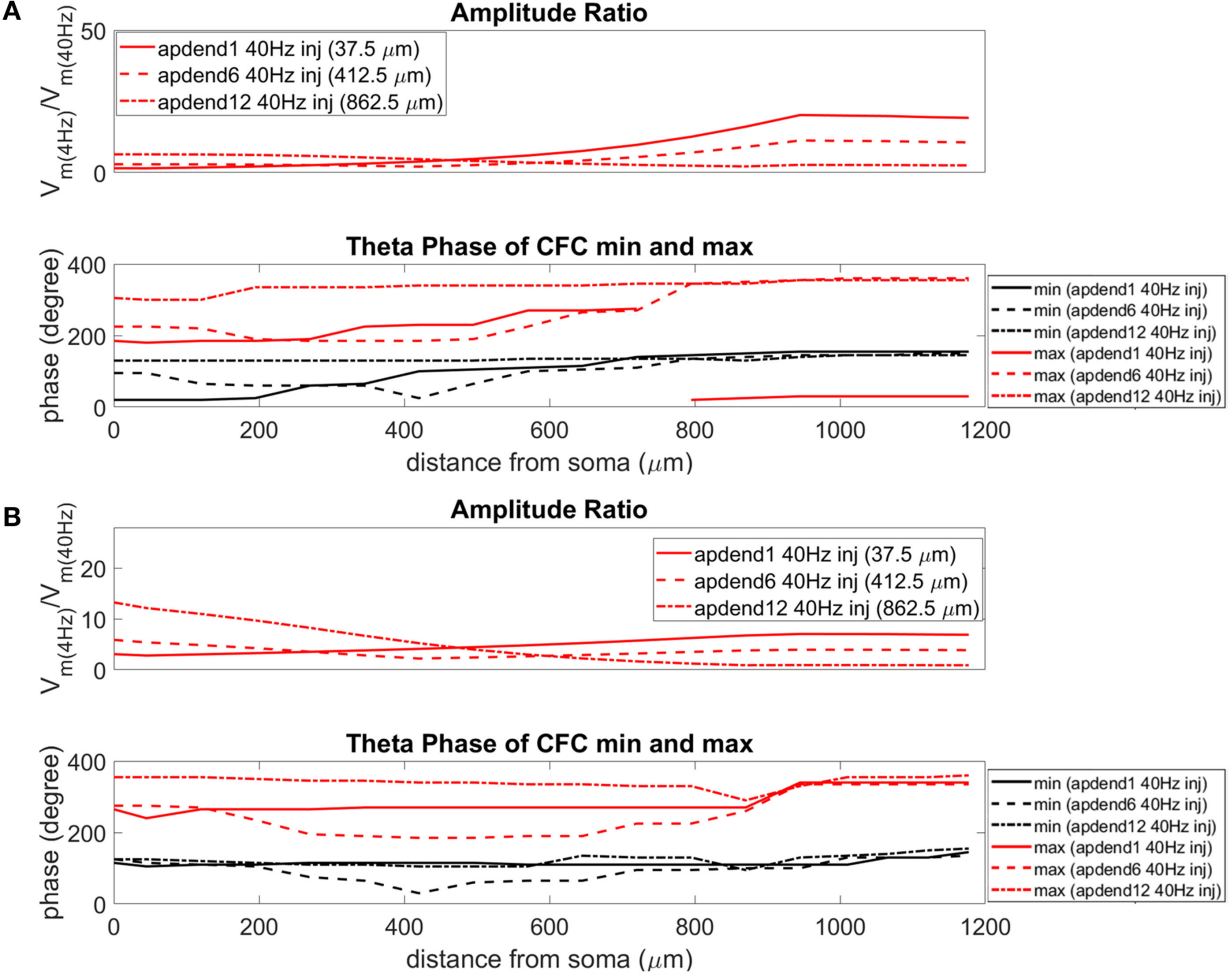
Amplitude ratio and theta phase of CFC for model with uniform I_h_ conductance. **(A)** Distal 4 Hz modulation, amplitude ratio (top) and theta phase (bottom) of minimum (black) and maximum (red) amplitude of 40 Hz membrane potential oscillation. Forty Hertz current injected into base of apical dendrite (solid lines), middle of apical dendrite (dashed lines), and distal apical dendrite (dot-dashed lines). **(B)** Perisomatic 4 Hz modulation, amplitude ratio (top), and theta phase (bottom) of minimum (black) and maximum (red) amplitude of 40 Hz membrane potential oscillation. Forty Hertz current injected into base of apical dendrite (solid lines), middle of apical dendrite (dashed lines), and distal apical dendrite (dot-dashed lines).

**Figure 8:**
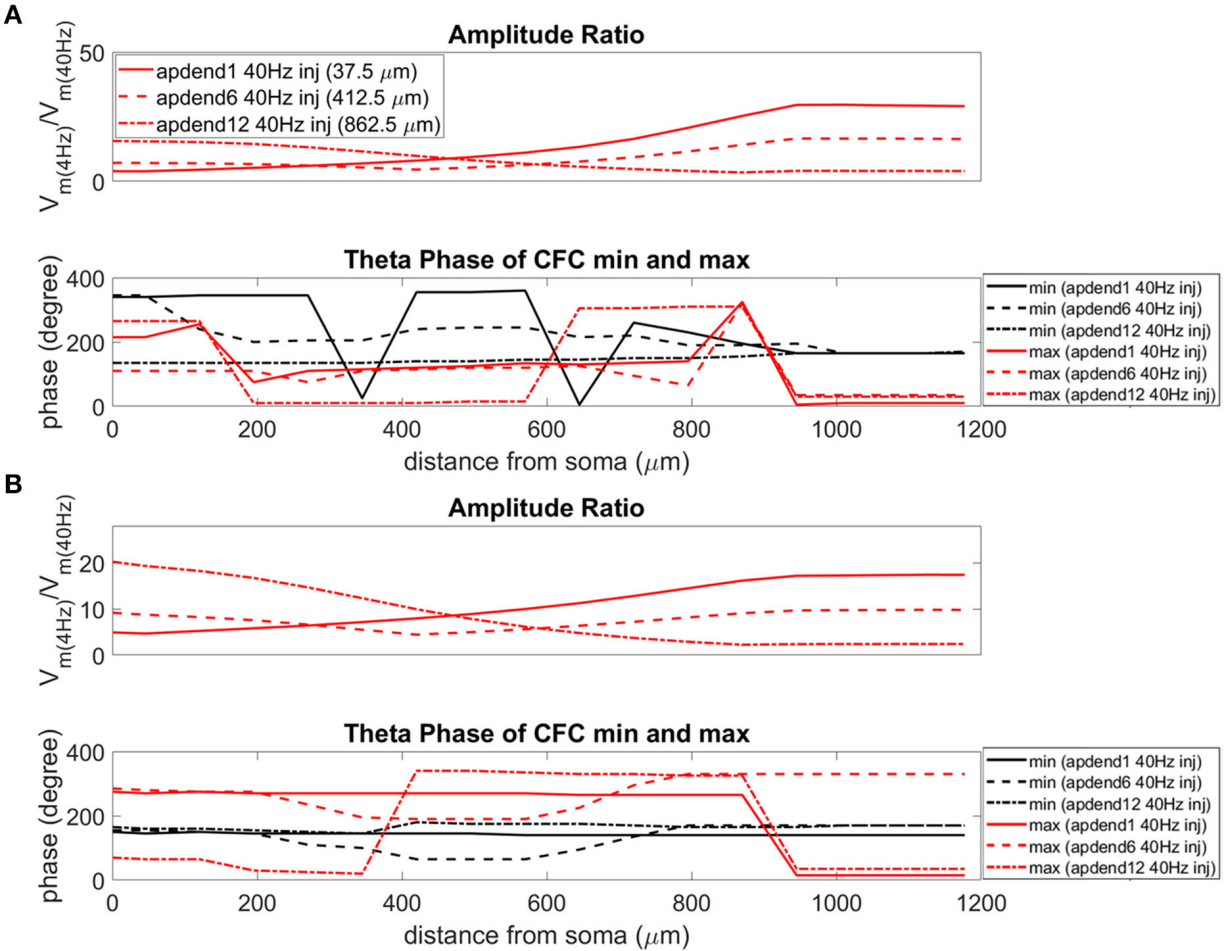
Amplitude ratio and theta phase of CFC for model with no I_h_ conductance. **(A)** Distal 4 Hz modulation, amplitude ratio (top), and theta phase (bottom) of minimum (black) and maximum (red) amplitude of 40 Hz membrane potential oscillation. Forty Hertz current injected into base of apical dendrite (solid lines), middle of apical dendrite (dashed lines), and distal apical dendrite (dot-dashed lines). **(B)** Perisomatic 4 Hz modulation, amplitude ratio (top), and theta phase (bottom) of minimum (black) and maximum (red) amplitude of 40 Hz membrane potential oscillation. Forty Hertz current injected into base of apical dendrite (solid lines), middle of apical dendrite (dashed lines), and distal apical dendrite (dot-dashed lines).

The locations of the current injections for the distal 4 Hz modulation scenario are presented in Figure 4A, and the profiles of height ratio along the soma-apical dendrite axis for models with I_h_ conductance densities configured for a soma-apical dendrite exponential gradient, uniform distribution, and zero conductance are presented in Figures 4B–D, respectively. (For clarity of presentation, the height ratio profiles in Figures 4B–D, 5B–D, and MI profiles in Figures S1B–D, S2B–D, were not calculated as continuous functions of distance from the soma, rather they were computed for each compartment which is shown in relation to distance from the soma in part A of each plot. The same format is used in **Figures 6–8**). All three I_h_ configurations show the same general pattern: strongest CFC in apical tuft (975–1,200 μm from the soma), with the soma and most of the lower apical dendrite (<700 μm from soma) exhibiting weaker CFC. For all three I_h_ configurations, the weakest CFC was observed between the soma and middle apical dendrite (0–450 μm from soma) when the 40 Hz current injection occurred in the soma or middle apical compartments. In particular, under these conditions in the model with either exponential gradient in I_h_ or no I_h_, CFC strength almost reduced to zero (height ratios <0.02). On the other hand, when the 40 Hz current injection occurred in the distal apical dendrite (12th apical dendrite compartment, 825 μm from the soma), CFC was strong throughout the soma-apical dendrite axis and tuft.

The strongest CFC occurred in the model with a uniform distribution in I_h_ conductance density (Figure 4C). When the exponential gradient was added to the model (Figure 4B), CFC strength was uniformly lower, by almost 50%. When I_h_ was removed from the model, CFC strength was also lower, with differences in both the shape and relationships among the profiles.

### CFC Strength: Perisomatic 4 Hz Modulation

The locations of the current injections for the perisomatic 4 Hz modulation scenario are presented in Figure 5A, and the profiles of height ratio along the soma-apical dendrite axis for models with I_h_ conductance densities configured for a soma-apical dendrite exponential gradient, uniform distribution, and zero conductance are presented in Figures 5B–D, respectively. All three I_h_ configurations again show a similar pattern for CFC strength. For compartments nearest the soma, CFC was strongest when the 40 Hz current injection occurred in distal apical dendrite, followed by 40 Hz current injection in middle apical dendrite, and finally weakest for 40 Hz current injection at the base of the apical dendrite (37.5 μm from soma). As you move up the apical dendrite toward the apical tuft, crossovers occur where now the strongest CFC was observed for 40 Hz current injection into the base of the apical dendrite, followed by injection into middle apical dendrite, and lastly injection into distal apical dendrite. When I_h_ is in the model and the 40 Hz current injection occurs in distal apical dendrite, CFC is almost completely eliminated at the site of the injection (12th apical dendrite compartment) (also see Figures S2B,C). On the other hand, CFC is almost completely eliminated in the middle apical compartments when I_h_ is not included in the model and the 40 Hz current injection occurs in the middle of the apical dendrite (see also Figure S2D).

Once again, CFC was strongest in the model with uniform I_h_ conductance density. CFC strength decreased by 30–40% when the exponential gradient in I_h_ conductance density was added to the model. Unlike in the previous injection scenario when the 4 Hz modulation occurred distally, when I_h_ was excluded from the model, there was a more noticeable decrease in CFC strength, particularly for the case when the 40 Hz current was injected into the base and middle of the apical dendrite.

## Amplitude and Phase Information

### Amplitude

The current injection amplitude sensitivity analysis presented in Figure 3 showed that there is greater dependence of CFC strength on the amplitude of the 4 Hz current injection than on the amplitude of the 40 Hz current injection. Because these two current injections differentially contribute to membrane potential oscillations throughout the model, it should be useful to compare the relative strength of membrane potential oscillations at 4 and 40 Hz to better understand CFC strength for any given compartment. The top panel in Figures 6A,B (exponential gradient I_h_), Figures 7A,B (uniform I_h_), and Figures 8A,B (no I_h_) show the *amplitude ratio* of membrane potential fluctuations at 4 Hz to the membrane potential fluctuations at 40 Hz [$\frac{V_{m\left(4Hz\right)}}{V_{m\left(40Hz\right)}}$] along the soma-apical dendrite axis and apical tuft. Because of the low-pass filtering properties of the membrane, this ratio is expected to be higher the greater the distance is between the 4 and 40 Hz current injections. Another condition in which this ratio is expected to be high is when both the 4 and 40 Hz current injection occurred on the same end of the soma-apical dendrite axis and have traveled the length of this axis to the opposite end. In both of these situations, the 40 Hz signal experiences a greater degree of filtering than the 4 Hz signal due to the passive and active membrane properties. To see separately the filtered 4 and 40 Hz components of membrane potential oscillations for the simulation runs of this study, see Figures S3, S4.

When the 4 Hz current was injected in distal apical dendrite (Figures 6A, 7A, 8A, top), *amplitude ratios* were highest in distal apical dendrite and apical tuft when there was distance between the 4 and 40 Hz current injections, i.e., when the 40 Hz current was injected in the base or middle of the apical dendrite. On the other hand, when the 40 Hz current injection also occurred distally, the *amplitude ratio* was highest in the soma yet still lower than the case of 40 Hz current injected in the base or middle of the apical dendrite.

When the 4 Hz current was injected in the soma (Figures 6B, 7B, 8B, top), *amplitude ratios* were highest in perisomatic compartments when there was distance between the 4 and 40 Hz injections (40 Hz injections in middle and distal apical dendrite). When the 40 Hz injection occurred in the base of the apical dendrite, the amplitude ratio was highest in distal apical regions.

### Phase

The bottom panel in Figures 6A,B (exponential gradient I_h_), Figures 7A,B (uniform I_h_), and Figures 8A,B (no I_h_) show the phase of the theta cycle that both the maximum and minimum amplitudes of the 40 Hz oscillation occur along the soma-apical dendrite axis and apical tuft. When I_h_ is present, minimum amplitudes for the 40 Hz oscillation occurred consistently within the first half of the theta cycle (20–130°) while maximum amplitudes for the 40 Hz oscillation occurred consistently within the second half of the theta cycle (180–340°). In addition, there tends to be a shift toward earlier theta phases that both the minimum and maximum 40 Hz oscillation amplitude occurs. This shift occurs in the lower half of the apical dendrite when the 40 Hz injection occurs there. This effect was the strongest (>100° shift to earlier theta phase) when the 40 Hz current was injected in the middle of the apical dendrite. In addition, this phase shift was very consistent because it occurred no matter the location of the 4 Hz modulation and for both exponential gradient and uniform I_h_ conductance densities—Figures 6A,B bottom and Figures 7A,B bottom, respectively. The phase shift that occurred when the 40 Hz current was injected into the base of the apical dendrite was more variable, being drastically reduced when the 4 Hz modulation occurred in distal apical dendrite (Figure 6A bottom and Figure 7A bottom).

When I_h_ is removed from the model, the phase relationship between minimum and maximum 40 Hz oscillation amplitude fundamentally changes (Figures 8A,B bottom). In the case of distal 4 Hz modulation, the phase relationship almost entirely flips so that the maximum 40 Hz oscillation amplitude often times occurred in the first half of the theta cycle while minimum 40 Hz oscillation amplitude occurs in the second half of the theta cycle. Whether the 4 Hz modulation occurs distally (Figure 8A bottom) or perisomatically (Figure 8B bottom), the phases of the minimum and maximum 40 Hz oscillation amplitude occur much closer together near the middle of the theta cycle relative to when the I_h_ conductance is in the model.

## Discussion

### Implications on the Circuitry Underlying CFC

Distal 4 Hz modulation produced the strongest CFC in the distal regions of the model regardless of the location of the 40 Hz injection. In particular, when I_h_ was present, whether uniformly or exhibiting an exponential gradient, CFC was strongest for distal 4 Hz modulation and 40 Hz injection into the base and middle of the apical dendrite. On the other hand, when the 4 Hz modulation occurred in the soma, each model saw a significant reduction in the maximum strength of CFC.

The injection scenario of distal 4 Hz modulation and 40 Hz current injection in the base and middle of the apical dendrite is similar to what is typically believed to be a common input pattern to large cortical pyramidal neurons, such as theta or alpha matrix thalamocortical input to distal apical dendrite and tuft, and gamma core thalamocortical input in the perisomatic region (VanRullen and Koch, 2003; Spruston, 2008; Hawkins and Ahmad, 2016). Our results suggest that the processing of this type of input to large cortical pyramidal neurons could be a source of CFC observed within the cortex.

The different I_h_ conductance density distributions used in our models impacts each model's input resistance to the 4 Hz modulation (Table 2). Input resistance increases as the I_h_ conductance density decreases, which happens when the distribution is changed from the exponential gradient to the uniform distribution. Input resistance increases further when I_h_ is removed. The effect of higher input resistance is larger membrane potential oscillations at 4 Hz in response to the 4 Hz modulation. Therefore, the exponential gradient in I_h_ more effectively filters the 4 Hz signal, leading to smaller *amplitude ratios* for this model than for the model with uniform I_h_. Likewise, removing I_h_ lead to the highest *amplitude ratios*. As suggested by Figure 3, the profiles of *amplitude ratio* for the exponential gradient I_h_ model and uniform I_h_ model are a good indicator of CFC strength. In general, the higher the amplitude ratio, the stronger the CFC. However, for the model with no I_h_, this trend did not continue. In this case, the highest *amplitude ratios* less faithfully produced the strongest CFC. Furthermore, despite producing the highest amplitude ratios seen in this study, the model with no I_h_ produced CFC that was comparable in strength to that produced by the exponential gradient I_h_ model when 4 Hz modulation was distal, and weaker CFC when 4 Hz modulation occurred perisomatically. This suggests that the *amplitude ratio* is only a good predictor of CFC strength when the I_h_ conductance is present.

**Table 2:** Input resistance (MΩ) for the compartments receiving the 4 Hz injection (soma and apdend13) for all models used in this study.

	**No I_h_**	**Uniform I_h_**	**Exp gradient I_h_**
perisomatic modulation (4 Hz, soma)	54	34	25
distal modulation(4 Hz, apdend13)	76	52	30

*Input resistance obtained by dividing the amplitude of the filtered 4 Hz membrane potential oscillation for a compartment by the amplitude of the 4 Hz current injected into the compartment (1.5 nA)*.

### Implications for Experimentalists

CFC has been observed for a wide range of spatiotemporal scales of brain activity, such as intracellular, LFP, and EEG recordings (Tort et al., 2010; Hyafil et al., 2015). The CFC profiles that we have observed for our current injection scenarios of distal 4 Hz modulation with perisomatic and middle apical dendrite 40 Hz injection is compatible with CFC results obtained by Sotero et al. (2015) who obtained LFP measurements at 100 μm intervals throughout the cortical depth in rats and performed CFC analysis on the signals. In our simulations, the 40 Hz oscillation generally was maximum during the hyperpolarizing phase of the 4 Hz oscillation and was minimum during the depolarizing phase of the 4 Hz modulation. A similar phase relationship between the 4 and 40 Hz oscillation was observed in Sotero et al. (2015). The results of the simulations in our study and the results of experimental work further suggests that the processing of distal slow input and perisomatic fast input by large cortical pyramidal neurons could be an underlying contributor to the CFC observed by meso- and macro-scale measurements, such as LFP and EEG.

**Figure 9:**
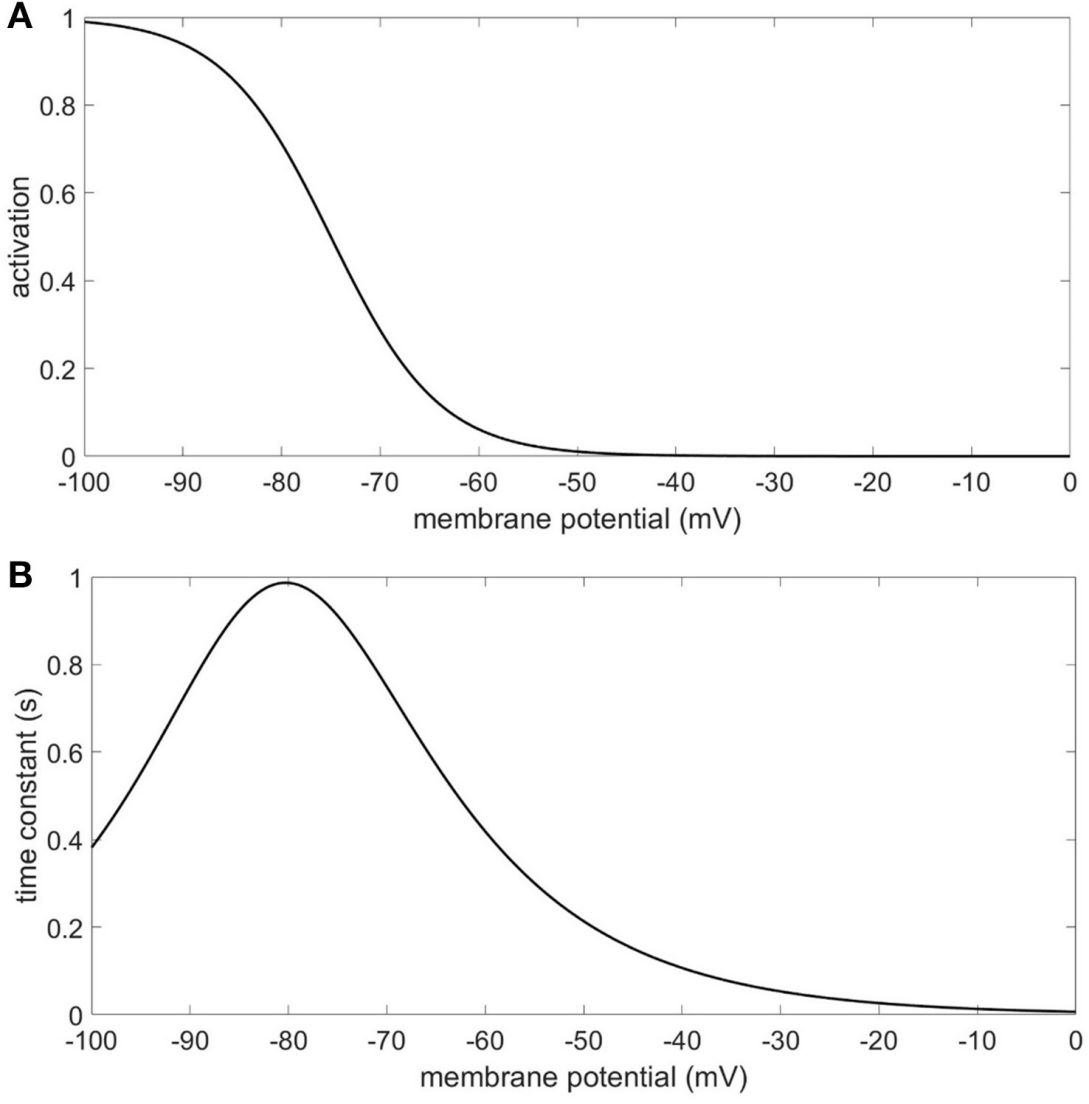
I_h_ conductance **(A)** activation function **(B)** time constant.

Due to the impact of the I_h_ conductance on resting membrane potential, a compartment's resting membrane potential depends on where it is in the model and on which model it is in. The range of resting membrane potentials for the compartments in this study was −85 to −56 mV. The 4 Hz modulation starting from resting membrane potential will alternately activate and deactivate the I_h_ conductance [modeled after the anomalous rectifier in Traub et al. (2003, 2005)] to varying degrees depending on the compartment and I_h_ configuration (Figure 9). We observed that the presence of the I_h_ conductance in our models not only modulated the strength of CFC, it also imposed order in the phase characteristics of CFC. The phase information in the results of our simulations suggest that the amplitude of the 40 Hz oscillation is minimized when the I_h_ conductance is deactivating. This occurs during the depolarizing phase of the 4 Hz modulation which raises the membrane potential of the neuron and reduces the activation of the I_h_ conductance. On the other hand, our results show that the amplitude of the 40 Hz oscillation is maximized when the I_h_ conductance is activating, which occurs during the hyperpolarizing phase of the 4 Hz modulation. Removing the I_h_ conductance often resulted in an inverted and much more variable relationship between maximum 40 Hz membrane potential oscillation and minimum 40 Hz membrane potential oscillation, suggesting that the activation and deactivation of the I_h_ conductance plays a critical role in producing the CFC seen in our simulations. There are other ionic conductances included in our model that can, along with the I_h_ conductance, contribute to the occurrence of CFC. However, our results indicate that the I_h_ conductance can significantly moderate the strength of CFC and impose order on the timing of the modulation experienced by the faster signal.

### Connection to Epilepsy Research

Large pyramidal neurons in cortical layer five and the hippocampus are known to have an exponentially-increasing gradient in the conductance density for the I_h_ conductance along the soma-apical dendrite axis (Hu et al., 2009; Nusser, 2009; Hay et al., 2011). It has been reported that when these neurons lack this exponential gradient, the result is that these neurons may become epileptic sources, at least in part because of increased excitability of these neurons (Brennan et al., 2016). In our study, we have seen that a lack of this exponential gradient is also associated with increased CFC. Therefore, epilepsy may be impacted by not only increased excitability in the underlying neurons, but also increased CFC within these neurons.

## Conclusion

We take a detailed look at the possible contribution of individual pyramidal neurons to CFC. Large pyramidal neurons, such as neocortical layer five or hippocampal pyramidal neurons, play a central role in the functioning of neocortical and limbic microcircuits. The neuronal membrane of these neurons has a large spatial extent and different parts of the neuron (e.g., proximal vs. distal) receive inputs from different populations of neurons within both local and widespread circuitry. Our study examined the ways in which different configurations of simultaneous fast and slow input are processed by individual pyramidal neurons and interact with each other to produce CFC. Furthermore, we have identified I_h_ as a current that has a large impact on the occurrence of CFC within pyramidal neurons.

This study can potentially shed light on which configurations of fast and slow input to pyramidal neurons produce the strongest CFC, and where exactly within the neuron CFC is strongest under realistic conditions of input. In addition, this study can illuminate the reasons why there may be differences between CFC strength observed in different regions of the brain and between different populations of neurons based on the configuration of the I_h_ conductance. This type of analysis may help experimentalists discriminate between CFC that originates from different underlying physiological mechanisms or determine if an exponential gradient in I_h_ conductance density is present or not.

## Data Availability Statement

The raw data supporting the conclusions of this article will be made available by the authors, without undue reservation.

## Author Contributions

All authors contributed equally to experimental design and theoretical considerations. AY helped establish single-neuron model and implemented all simulated current injection capabilities. MF ran all simulations and developed CFC analysis post-processing algorithms. All authors contributed to data interpretation and manuscript preparation.

## Conflict of Interest

The authors declare that the research was conducted in the absence of any commercial or financial relationships that could be construed as a potential conflict of interest.
