# Supplementary material for: Assessing the Impact of Ih Conductance on Cross-Frequency Coupling in Model Pyramidal Neurons
Source: Front Comput Neurosci. 2020 Sep 10;14:81. doi: 10.3389/fncom.2020.00081 (PMC7511577; doi:10.3389/fncom.2020.00081)
Supplement: Supplementary file 1 [file Data_Sheet_1.docx]

**SUPPLEMENTAL RESULTS**

CFC STRENGTH: MODULATION INDEX (MI)

Distal 4 Hz modulation


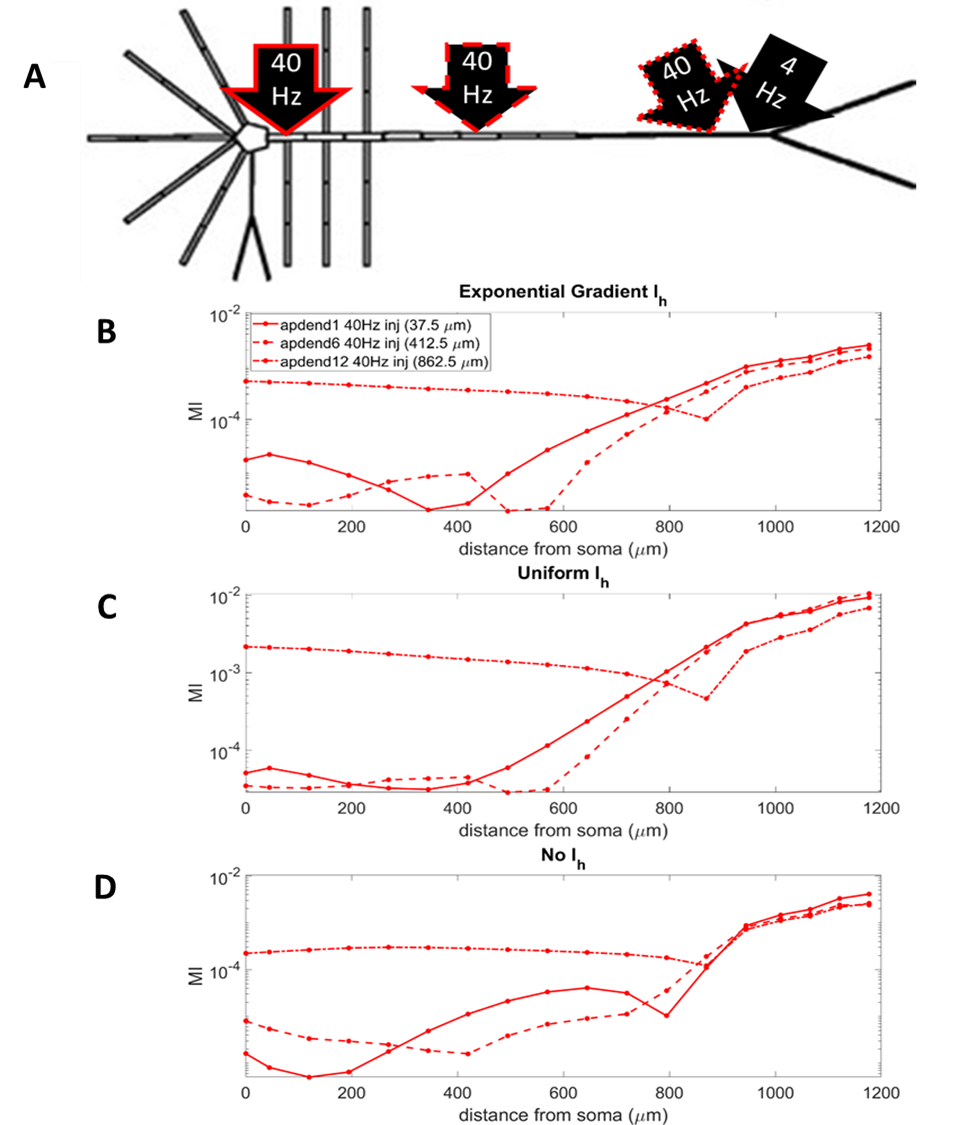


Figure S1 (A) Distal apical dendrite 1.5 nA, 4 Hz modulation (black arrow) with 1.5 nA 40 Hz current injections in base of apical dendrite (black arrow, solid red outline), middle apical dendrite (black arrow, dashed red outline), and distal apical dendrite (black arrow, dot-dashed red outline). (B) Modulation index (MI) calculated for the soma, apical dendrite, and apical tuft in the model with exponential gradient in I_h_ conductance density along apical dendrite. (C) MI calculated for the soma, apical dendrite, and apical tuft in model with uniform I_h_ conductance density. (D) MI calculated for the soma, apical dendrite, and apical tuft in model with no I_h_ conductance. Dot symbols along the profiles in B-D indicate the distance from the soma of compartments along the soma-apical dendrite axis and apical tuft (measured from the beginning of each compartment).

Perisomatic 4 Hz modulation


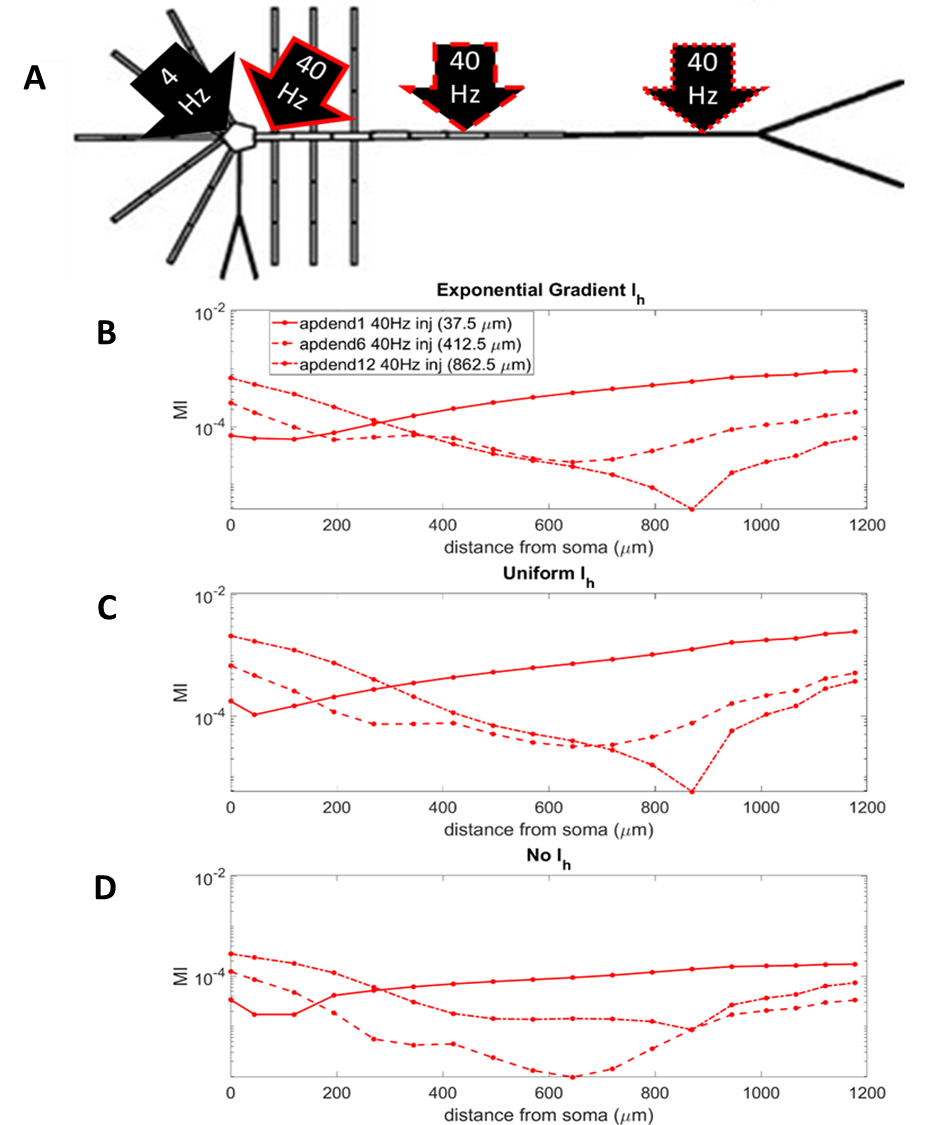


Figure S2 (A) Perisomatic 1.5 nA, 4 Hz modulation (black arrow) with 1.5 nA 40 Hz current injections in base of apical dendrite (black arrow, solid red outline), middle apical dendrite (black arrow, dashed red outline), and distal apical dendrite (black arrow, dot-dashed red outline). (B) MI calculated for the soma, apical dendrite, and apical tuft in model with exponential gradient in I_h_ conductance density along apical dendrite. (C) MI calculated for the soma, apical dendrite, and apical tuft in model with uniform I_h_ conductance density. (D) MI calculated for the soma, apical dendrite, and apical tuft in model with no I_h_ conductance. Dot symbols along the profiles in B-D indicate the distance from the soma of compartments along the soma-apical dendrite axis and apical tuft (measured from the beginning of each compartment).

AMPLITUDE OF MEMBRANE POTENTIAL OSCILLATIONS

Distal 4Hz modulation


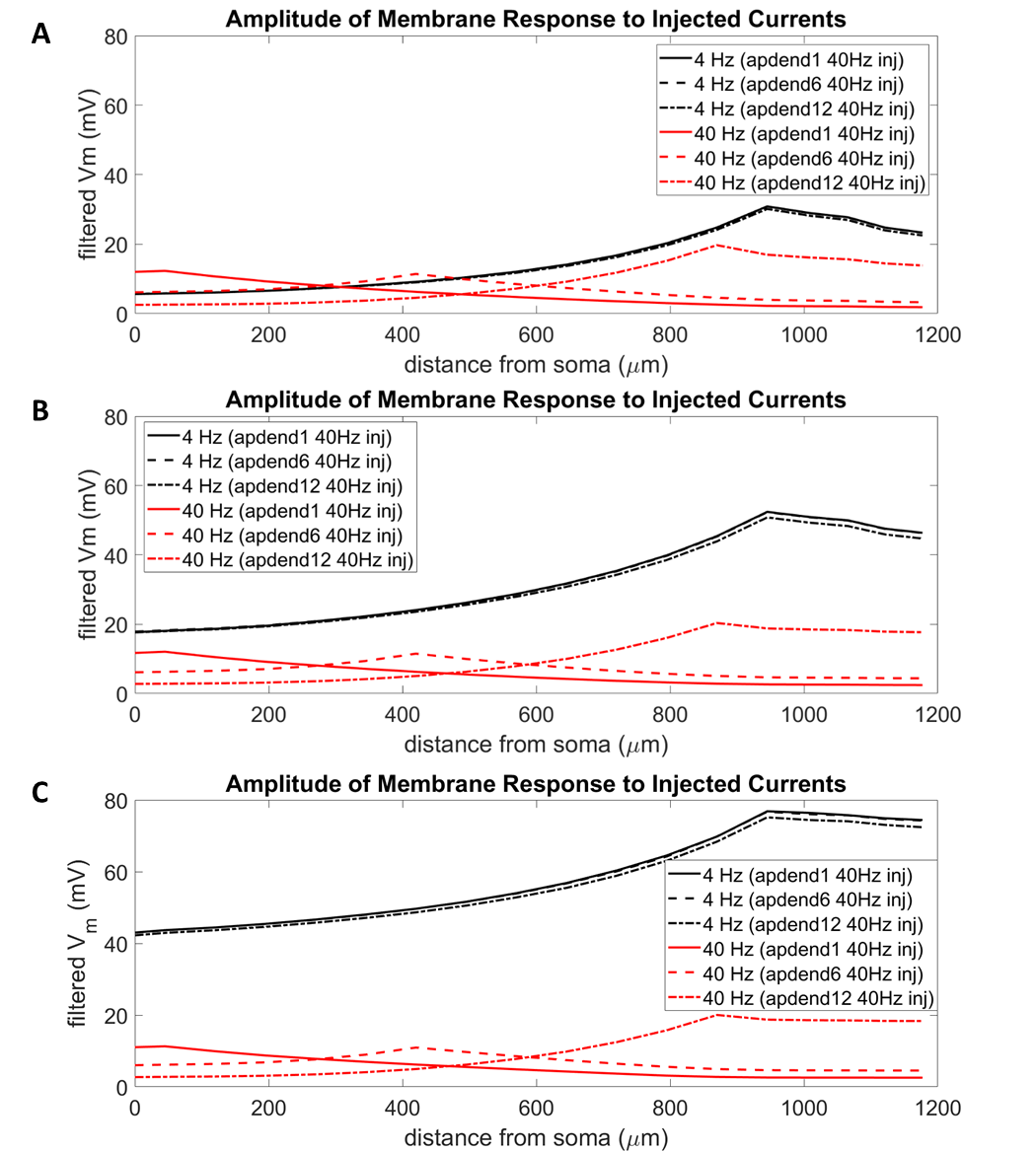


Figure S3 4 Hz (black) and 40 Hz (red) component of membrane potential oscillations for all injection scenarios (see legends) with distal 4 Hz modulation. (A) soma-apical dendrite exponential gradient in I_h_ conductance density. (B) uniform distribution in I_h_ conductance density. (C) no I_h_ conductance.

Perisomatic 4Hz modulation


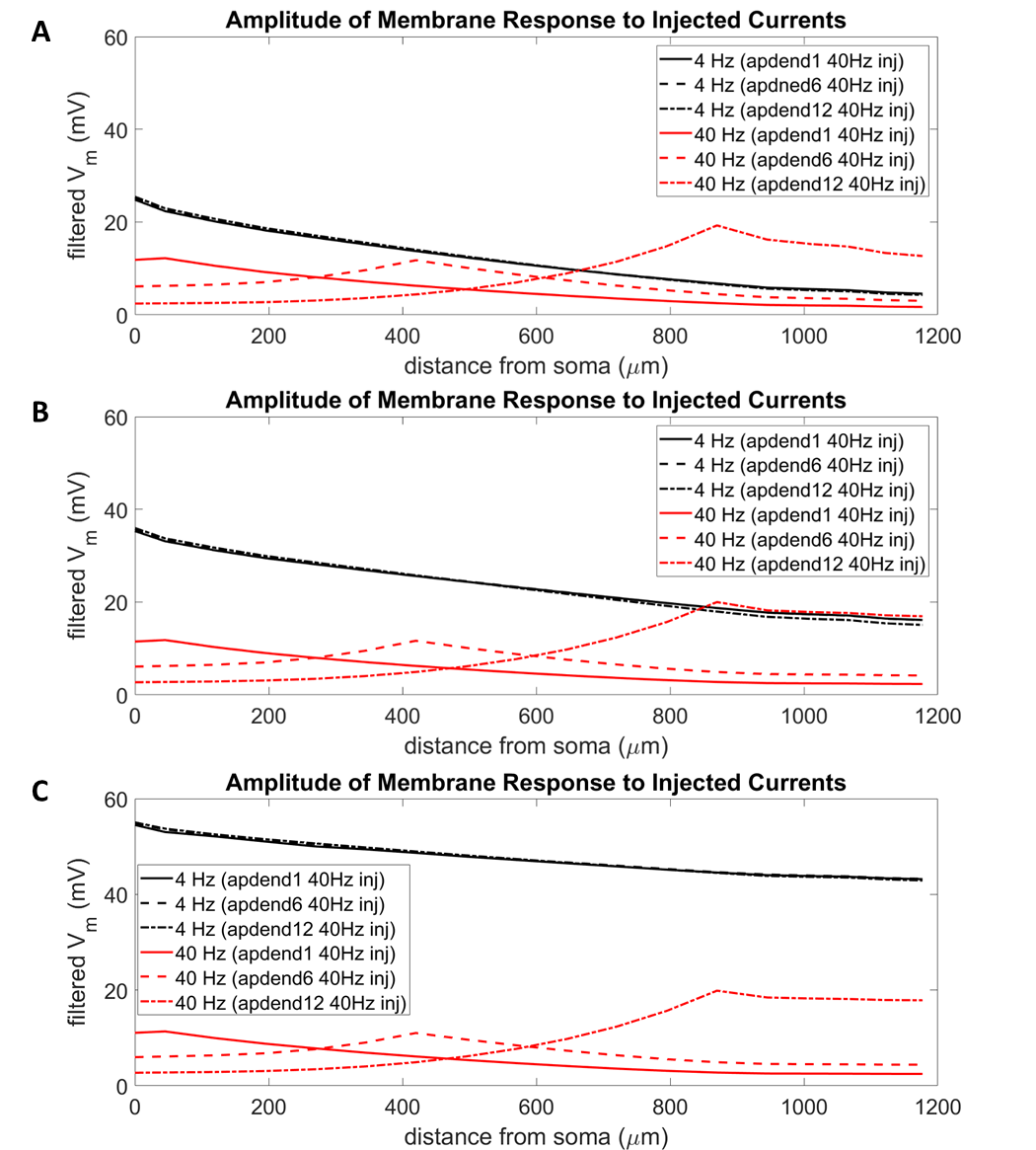


Figure S4 4 Hz (black) and 40 Hz (red) component of membrane potential oscillations for all injection scenarios (see legends) with perisomatic 4 Hz modulation. (A) soma-apical dendrite exponential gradient in I_h_ conductance density. (B) uniform distribution in I_h_ conductance density. (C) no I_h_ conductance.
